# Infants Born to Mothers With a New Coronavirus (COVID-19)

**DOI:** 10.3389/fped.2020.00104

**Published:** 2020-03-16

**Authors:** Yan Chen, Hua Peng, Lin Wang, Yin Zhao, Lingkong Zeng, Hui Gao, Yalan Liu

**Affiliations:** ^1^Department of Pediatric, Union Hospital, Tongji Medical College, Huazhong University of Science and Technology, Wuhan, China; ^2^Department of Obstetrics, Union Hospital, Tongji Medical College, Huazhong University of Science and Technology, Wuhan, China; ^3^Department of Neonatal, Wuhan Children's Hospital (Wuhan Maternal and Child Healthcare Hospital), Tongji Medical College, Huazhong University of Science and Technology, Wuhan, China

**Keywords:** newborns, clinical course, China, COVID-19 infected mothers, vertical transmission

## Abstract

A novel viral respiratory disease caused by severe acute respiratory syndrome coronavirus 2 (SARS-CoV-2), is responsible for an epidemic of the coronavirus disease 2019 (COVID-19) in cases in China and worldwide. Four full-term, singleton infants were born to pregnant women who tested positive for COVID-19 in the city of Wuhan, the capital of Hubei province, China, where the disease was first identified. Of the three infants, for who consent to be diagnostically tested was provided, none tested positive for the virus. None of the infants developed serious clinical symptoms such as fever, cough, diarrhea, or abnormal radiologic or hematologic evidence, and all four infants were alive at the time of hospital discharge. Two infants had rashes of unknown etiology at birth, and one had facial ulcerations. One infant had tachypnea and was supported by non-invasive mechanical ventilation for 3 days. One had rashes at birth but was discharged without parental consent for a diagnostic test. This case report describes the clinical course of four live born infants, born to pregnant women with the COVID-19 infection.

## Introduction

The new coronavirus 2019 (COVID-19) is an epidemic in Wuhan and the population is believed to be immunologically naïve. As the epidemic progresses, there remains little understanding of infant and childhood COVID-19 infections and their clinical picture. As of 22 February 2020, 77,043 cases of novel COVID-19 infections have been confirmed and 2,445 people have died (http://2019ncov.chinacdc.cn/2019-nCoV/). During this epidemic, four live-born infants were born in our medical center, to pregnant women with the COVID-19 infection. Three of the four pregnant women gave birth by cesarean section due to concerns about symptomatic maternal infection. The other infant was born by vaginal delivery to a mother experiencing fever (highest temperature 38.3°C), with a diagnostically confirmed infection. The most important question is whether the COVID-19 could be transmitted vertically to the fetus from the pregnant mother and cause a clinically significant infection. Recently, a finding from nine other cases suggested that there is no evidence for intrauterine infection caused by vertical transmission in women who develop COVID-19 pneumonia in late pregnancy (1). We believe this present report is the second case report on vertical transmission between COVID-19 pregnant women and their infants. Moreover, this report will focus more on infants. This case report describes the clinical course of four live born infants born to pregnant women with the COVID-19 infection.

## Case Report

### Cases of the Mothers

All four mothers were symptomatically infected with COVID-19 during the 3rd trimester. On admission, the regular symptoms of pregnant mothers with COVID-19 were fever (three out of four patients), cough (two out of four patients), myalgia or fatigue (two out of four patients), and headache (two out of four patients). Only one patient felt reduced fetal movement and one experienced dyspnea. Lymphocytes were below the normal range (lymphocyte count <1.1 × 10^9^/L) in all patients, and two patients showed lymphopenia (lymphocyte count <1.0 × 10^9^/L). Both leucocytes and platelet counts were below the normal range (white blood cell count <4 × 10^9^/L, platelet count <100 × 10^9^/L) in the mother in Case 4 (shown in Table 1). The mother in Case 3 developed anemia (hemoglobin 83 g/L) and dyspnea 5 days after being admitted. There was a significant increase of the level of C-response protein in all pregnant mothers. Coagulation function and blood biochemistry of all the mothers were normal. Five respiratory pathogens (Mycoplasma, Chlamydia, Respiratory syncytial virus, Adenovirus, and Coxsackie virus) and the nucleic acid of influenza viruses A and B of all patients were negative (laboratory findings shown in Table 1). An RT-PCR assay confirmed that the throat swab of the four pregnant women were positive for COVID-19. Abnormalities in chest CT images and bilateral involvement were detected among all pregnant women. A cesarean section was performed for three patients in the acute phase of the disease while one patient underwent vaginal delivery because of the onset of labor. Four full-term infants were born. All infants were isolated from their mother immediately after birth. We describe the clinical course of these four infants (laboratory findings shown in Table 2). Three mothers of the infants recovered from their COVID-19 infections and were released 3–5 days after delivery. However, one mother suffered severe dyspnea after delivery which required respiratory support—she did, however, survive. All four infants and their mothers were healthy upon a post-discharge follow-up.

**Table 1:** Clinic and laboratory characteristics of mothers.

	**Case 1**	**Case 2**	**Case 3**	**Case 4**
Age, years	28	34	23	31
Maternal history	G1P1	G1P1	G2P1	G2P2
Comorbidity	No	Cholecystitis	Placenta previa	No
**Signs and symptoms**				
Fever	Yes	No	Yes	Yes
Cough	Yes	No	Yes	No
Fatigue	Yes	No	No	Yes
Headache	Yes	No	No	Yes
Mulligrubs	No	Yes	No	No
Dyspnea	Yes	No	Yes	No
Fetal movement	Normal	Normal	Normal	Decrease
Heart rate (per min, at arrival)	117	99	122	137
**Laboratory findings**				
White blood cell count, × 10^9^/L	4.7	6.8	8.3	3.7
Lymphocyte count, × 10^9^/L	1.0	1.1	0.8	0.7
Neutrophil count, × 10^9^/L	3.5	5.3	7.1	2.8
Hemoglobin, g/L	111	123	124	131
Platelet count, × 10^9^/L	218	160	114	92
C-response Protein, mg/L	11	26	51	19
Activated partial thromboplastin time, s	53.1	40.2	54	37.3
Prothrombin time, s	13.1	11.5	12.6	11.9
D-dimer, mg/L	1.9	2.2	3.0	0.6
FIB	1.5	4.8	4.9	3.8
Albumin, g/L	32.9	30.8	33	33.1
Alanine aminotransferase, U/L	25	21	19	22
Aspartate aminotransferase, U/L	37	29	27	13
Total bilirubin, mmol/L	10.6	10	16.4	10.8
Creatinine, μmol/L	46.0	44.9	39.9	57.2
Blood urea nitrogen, mmol/L	2.8	2.2	1.5	3.0
Seven respiratory pathogen^@^	Neg	Neg	Neg	Neg

@ *Seven respiratory pathogen: Mycoplasma, Chlamydia, Respiratory syncytial virus, Adenovirus, Coxsackie virus, and influenza viruses A/B*.

**Table 2:** Clinic and laboratory characteristics of the newborns.

	**Case 1**	**Case 2**	**Case 3**	**Case 4**
2019-nCoV of father	Neg	Neg	Neg	Positive
Sex	female	male	male	male
Gestational age, weeks	37^+2^	39^+0^	37^+3^	38^+4^
Birth weight, g	3,200	3,050	3,800	3,550
Apgar score (1, 5 min)	8.9	8.9	7.8	8.9
Delivery mode	Cesarean	Cesarean	Cesarean	Vaginal delivery
**Laboratory findings**				
White blood cell count, × 10^9^/L	–	13.1	18.7	22.2
Lymphocyte count, × 10^9^/L	–	2.6	5.2	2.7
Neutrophil count, × 10^9^/L	–	9.0	11.6	7.5
Hemoglobin, g/L	–	198	172	175
Platelet count, × 10^9^/L	–	217	258	285
C-response protein, mg/L	–	1	0	0
Albumin, g/L	–	29	35.9	42.6
Alanine aminotransferase, U/L	–	6	10	28
Aspartate aminotransferase, U/L	–	23	41	116
Total bilirubin, mmol/L	–	41.7	43.1	80.2
Activated partial thromboplastin time, s	–	15.5	14.3	13.6
Prothrombin time, s	–	50.8	52.3	53
INR	–	1.3	1.1	1.1
FIB	–	3.7	2.2	1.6
**Signs and symptoms**				
Dyspnea	No	No	Yes	No
Oxygen therapy	No	No	Yes	No
Heart rate	Normal	Normal	Normal	Normal
Blood pressure	Normal	Normal	Normal	Normal
Edema	No	Yes	Yes	No
Rash	No	Yes	Yes	No
Chest radiograph	–	Normal	TTN	Normal
2019-nCoV (throat swab)	Neg	–	Neg	Neg

### Cases of the Infants

Three male, and one female infant was born beyond 37 weeks' gestation and had a birthweight above 3,000 g. All infants had a 1-min Apgar score of 7–8 and 5-min Apgar score of 8–9 (Table 2). They were isolated from their mothers immediately after birth and received formula feeding. Three of the four infants tested negative for COVID-19 using a throat swab specimen in RT-PCR 72 h after birth and one baby's parents did not provide consent for their baby to be tested for COVID-19.

Two of the four infants were healthy. Two of the four infants had rashes after birth, however, the rash distribution and shape differed. The infant in Case 2 had some maculopapules scattered all over the body, and one facial skin ulceration on the forehead (size about 0.3 × 0.5 cm^2^). The rash disappeared and skin desquamation appeared the next day without any treatment. The rash of the infant in Case 3 was present on the forehead and seemed to diffuse small miliary red papules on day 2. The rash disappeared on day 10 without treatment (Table 2). The infant in Case 2, the mother of whom had cholecystitis, developed edema of the lateral thigh on day 3, and the level of serum albumin was only 26 g/L. The baby was taking full formula feeds on day 4. The baby was discharged from the NICU (neonatal intensive care unit) 6 days after birth. The infant in Case 3, the mother of whom had placenta previa, suffered transient tachypnea of the newborn (TTN) and required nasal- Continuous Positive Airway Pressure (nCPAP) after birth. Breathing became regular within 3 days. The baby was taking full formula feeds on day 5 and was discharged from the NICU on day 7 (Table 2).

## Discussion

In this study, four pregnant women were confirmed to have the COVID-19 infection. One mother experienced reduced fetal movement. One mother developed anemia and dyspnea after admission. Of the three infants whose parents provided consent to be diagnostically tested, none tested positive for the virus. None of the infants developed serious clinical symptoms such as fever, cough, or diarrhea. Two newborns had a rash, which disappeared spontaneously without treatment; one newborn had mild dyspnea, and was considered to suffer from TTN and supported by non-invasive mechanical ventilation for 3 days. All of the four babies are doing well and have been formula feeding since birth.

Coronavirus (CoVs) (2) is an enveloped positive-sense RNA virus, which infects humans and a wide variety of animals, causing diseases in the respiratory, enteric, hepatic, and neurological systems with varying severity (3). In the past few decades, newly evolved CoVs have posed a global threat to public health, such as severe acute respiratory syndrome coronavirus (SARS-CoV) and Middle East respiratory syndrome coronavirus (MERS-CoV) that were implicated in the 2003 outbreak in Guangdong, China and the 2012 outbreak in the Middle East, respectively (2). On 10 January 2020, a new coronavirus causing a pneumonia epidemic in Wuhan City in central China was denoted as COVID-19 by the World Health Organization (WHO) (4). As of 22 February 2020, nearly 77,043 COVID-19 infections in humans have been confirmed in China, with at least 2,445 reported deaths. As reported herein, four pregnant women were confirmed to have the COVID-19 infection in our medical center, which is designated as one of the treatment centers for pregnant women with the COVID-19 infection. Importantly, we found neither SARS-CoV-2 diagnostic positivity nor immediate evidence of symptomatic COVID-19 among the infants born to the symptomatic, test-positive mothers.

On the basis of previous reports (5–7), SARS-CoV and MERS-CoV were associated with critical maternal illness, spontaneous abortion, or even maternal death. In these four pregnant women with the COVID-19 infection, three had fever, two had a cough and experienced headache. In laboratory data, there was lower lymphocyte count and higher CRP in blood analysis. Typical CT images of COVID-19 infection with ground glass changes were presented in these pregnant patients. These four pregnant women had no critical maternal illness. Only one of them experienced reduced fetal movement and one had dyspnea. These symptoms, at onset of delivery, were similar to other populations (8). To prevent COVID-19 intrauterine, perinatal, and postnatal transmission, three pregnant women received a cesarean section. One of the three pregnant women suffered placenta previa, which made it necessary to opt for a cesarean section. Only one pregnant mother adopted a vaginal delivery because of an emergency labor process.

Shek et al. (9) reported that perinatal transmission of the SARS-associated coronavirus was not detected in any of the five live born infants who were born to pregnant women with SARS during the community outbreak in Hong Kong in 2003. In addition, none of the infants developed clinical, radiologic, hematologic, or biochemical evidence suggestive of SARS. Consistent with these reports, in our study, RT-PCR assay confirmed that the throat swab of the three cases were negative for COVID-19. We regret that the infant in Case 2 did not have a COVID-19 diagnosis as the baby's guardian's did not provide consent.

Assiri et al. (7) reported five cases of pregnant women infected with MERS-CoV from Saudi Arabia, and all pregnancies were in the second or third trimester. Among the five pregnancies, two pregnant women died during their illnesses, two resulted in perinatal death (one pregnancy resulted in intrauterine fetal demise, and one infant died 4 h after an emergency cesarean delivery). It was reported that 12 pregnant women were diagnosed to have the SARS infection during the outbreak in Hong Kong (10). Seven mothers presented in the first trimester, and the rest were in their late second and third trimester. It was reported that the SARS infection in pregnant women could lead to severe intrauterine growth retardation, which could be due to the prolonged usage of high dose systemic corticosteroids or antiviral agents and/or the impact of a severe maternal debilitating illness on normal fetal growth (9, 10). In this study, all four cases reported on were delivered during the acute phase of the illness, at 37–39 weeks of gestation, and the birth weight of all the babies were appropriate for their gestational age. Throughout the clinical course, there were no manifestations or radiologic, hematologic, or biochemical evidence suggestive of COVID-19 infection. This study is similar to reports of SARS infection (9) (Table 2).

Coronaviruses cause respiratory and intestinal infections in animals and humans (11). For adult patients, the clinical manifestations of COVID-19 infection include fever, cough, shortness of breath, muscle ache, sore throat, diarrhea, and so on (11). The minority of patients showed severe and even fatal respiratory diseases such as acute respiratory distress syndrome. According to imaging examination, most patients showed bilateral pneumonia, multiple mottling, or ground-glass opacity. In this study, only the infant in Case 3 showed dyspnea and required oxygen therapy. A chest radiograph of the infant in Case 3 showed that the brightness of the left lung was slightly decreased, and the texture of the right lung was slightly blurred. His condition was relieved gradually after 3 days of nCPAP treatment.

It has been confirmed that COVID-19 gravely damages leucocytes, and could lead to multiple organ damage along with the respiratory system (12). In this study, blood assays of the three infant cases were normal, and all the blood cell counts and hemoglobin concentrations fluctuated within the normal reference range. It is worth noting that both Case 2 and Case 3 presented a transient skin rash after birth. Whether this was attributable to the maternal inflammatory toxin effect requires further study. At follow up, the four newborns were health and had grown on formula feeding.

This feature reveals that none of the four newborns of the mothers with COVID-19 developed COVID-19 infection. In this study, viral nucleic acid detection using real-time polymerase chain reaction (RT-PCR) remains, is taken as the standard of COVID-19 infection. A recent retrospective analysis in adults showed that the sensitivity of RT-PCR is 71% for COVID-19 infection (13). Therefore, the reliability of diagnostic testing should be further evaluated, especially in children. Another limitation of this report was the small number of cases, and imperfect clinic data. No COVID-19 vertical transmission was detected. Further studies for viral infection in placenta, amniotic fluid, neonatal blood, gastric fluid, and anal swab, and the viral depending receptor on children will be detected in future.

## Data Availability Statement

The datasets generated for this study are available on request to the corresponding author.

## Ethics Statement

The studies involving human participants were reviewed and approved by the Institutional Review Board of Union Hospital, Huazhong University of Science & Technology. Written informed consent to participate in this study was provided by the participants' legal guardian/next of kin. Written informed consent was obtained from the individual(s), and minor(s)' legal guardian/next of kin, for the publication of any potentially identifiable images or data included in this article.

## Author Contributions

YC and HP designed the study, drafted the initial manuscript, and reviewed and revised the manuscript. LW, HG, YZ, and LZ designed the data collection instruments, collected the data, and reviewed and revised the manuscript. YL designed the study, coordinated, and supervised data collection, and critically reviewed the manuscript for important intellectual content. All authors approved the final manuscript as submitted and agree to be accountable for all aspects of the work.

### Conflict of Interest

The authors declare that the research was conducted in the absence of any commercial or financial relationships that could be construed as a potential conflict of interest.
